# Sociodemographic Analysis of Suicide Rates Among Older Adults Living in Ecuador: 1997–2019

**DOI:** 10.3389/fpubh.2021.726424

**Published:** 2021-10-08

**Authors:** M. Isabela Troya, Rebekka M. Gerstner, Freddy Narvaez, Ella Arensman

**Affiliations:** ^1^School of Public Health, College of Medicine and Health, University College Cork, Western Gateway Building, Cork, Ireland; ^2^National Suicide Research Foundation, University College Cork, Cork, Ireland; ^3^National Undersecretary of Health Services From the Ministry of Public Health, Quito, Ecuador; ^4^Unit of Health, Manuela Saenz Administration. Municipality of the Metropolitan District of Quito, Quito, Ecuador

**Keywords:** suicide, fatal self harm, mortality, undetermined death, older adult, Ecuador, LAMIC, global mental health

## Abstract

**Background:** Despite most suicides occurring in low-and-middle-income countries (LAMICs), limited reports on suicide rates in older adults among LAMICs are available. In Ecuador, high suicide rates have been reported among adolescents. Little is known about the epidemiology of suicides among older adults in Ecuador.

**Aim:** To examine the sociodemographic characteristics of suicides among older adults living in Ecuador from 1997 to 2019.

**Methods:** An observational study was conducted using Ecuador's National Institute of Census and Statistics database from 1997 to 2019 in Ecuadorians aged 60 and older. International Classification of Diseases 10th Revision (ICD-10) (X60-X84)-reported suicide deaths were included in addition to deaths of events of undetermined intent (Y21-Y33). Sex, age, ethnicity, educational level, and method of suicide were analyzed. Annual suicide rates were calculated per 100,000 by age, sex, and method. To examine the trends in rates of suicide, Joinpoint analysis using Poisson log-linear regression was used.

**Results:** Suicide rates of female older adults remained relatively stable between 1997 and 2019 with an average annual percentage increase of 2.4%, while the male rates increased between 2002 and 2009, 2014 and 2016, and maintained relatively stable within the past 3 years (2017–2019). The annual age-adjusted male suicide rate was 29.8 per 100,000, while the female suicide rate was 5.26 per 100,000 during the study period. When adding deaths of undetermined intent, the annual male rate was 60.5 per 100,000, while the same rate was 14.3 for women. The most common suicide method was hanging (55.7%) followed by self-poisoning (26.0%). The highest suicide numbers were reported in urban districts, men, and those with lower education status.

**Conclusion:** This study contributes to building the baseline for further studies on suicide rates of older adults in Ecuador. Results highlight priority areas of suicide prevention. By examining suicide trends over 23 years, findings can help inform policy and future interventions targeting suicide prevention.

## Introduction

The WHO (Global Mental Health Action Plan) and United Nations (Sustainable Development Goals, Goal 3) recognize the importance of reducing rates of suicides, one of the major causes of premature mortality globally. Worldwide, over 800,000 lives are lost to suicide (1).

Despite close to 80% of all suicides occurring in LAMICs, limited research exists on trends and rates of suicides in LAMICs (1). Findings from Ecuador, a LAMIC, show that between 2001 and 2015, 13,024 lives were lost to suicide, representing a substantial number of the population with long-lasting impacts on those left behind (2). Evidence indicates that suicides among adolescents and young adults (aged 15 to 24) in Ecuador have been increasing over the past few years, with estimated annual suicide rates of 6.27–6.48 per 100,000 among young people aged 10–19 from 2000 to 2009 (3, 4). Another study calculated the overall age-adjusted suicide rates in Ecuador from 2001 to 2014 among all age groups and found this to be 7.1 per 100,000 with men being three times more likely to die by suicide when compared to women (2). The same study found the highest rates among teenagers aged 13–20, with annual rates of close to 120 per 100,000 (2).

While research shows that suicide rates among children and adolescents (aged 10–19) in Ecuador are among the highest worldwide (5), and men of all ages are at an increased risk of suicide, little is known about suicide trends and characteristics among Ecuadorian older adults. Worldwide, older adults represent the highest age group to die by suicide (1). Hopelessness, depression, social isolation, and loneliness are among the most common motivations for suicide among older adults (6, 7). With an increasingly aging population, it is important to know the key characteristics and potential risk factors of suicide among older adults to inform effective intervention programs and suicide prevention strategies. The objective of this study was to examine the sociodemographic characteristics of suicides among older adults living in Ecuador from 1997 to 2019.

## Methods

### Study Design and Setting

An ecological secondary-based study was conducted using nationwide data from January 1997 to December 2019 in Ecuador of all deaths registered within a national database: National Institute of Census and Statistics (INEC). This is a national study involving data from all 24 provinces in the Republic of Ecuador provided by the National Institute of Census and Statistics (8). As of 2019, the Republic of Ecuador has a population of 17.3 million according to projections based on its most recent (2010) census. Ecuador is composed of 24 provinces, which are divided into four regions: Highlands/Sierra, Amazon, Coast, and Insular region (9). The country's capital of Quito is the largest city with a population of 2.73 million, followed by the coastal city of Guayaquil with 2.70 million habitants. Over 11 million people, close to two-thirds of the Ecuadorian population (64%), live in urban districts, while the remaining 6.2 million (36%) live in rural districts (9). The majority of the population (71.9%) self-identifies themselves as “mestizos,” a term used to refer to people of mixed-race ancestry (Indigenous and White European), while the remaining population self-identifies as Afro-Ecuadorian (7.2%), Indigenous (7%), White (6.1%), and other (7.8%) (9, 10).

Population data were obtained from projections based on the 2010 Census of the National Institute of Census and Statistics (9), for each age group, sex, and province.

### Participants, Outcomes, Data Sources, and Measurement

Deceased individuals aged 60 years or older resided in Ecuador from January 1997 to December 2019. ICD-10 X60-X84 reported suicide deaths were included in addition to external deaths of undetermined intent Y10-Y34, registered in databases of the National Institute of Census and Statistics (8). In terms of the study outcomes, these were deaths by suicide ICD-10 (X60-X84) and external deaths of undetermined intent ICD Y10-Y34. The INEC provides a national database on mortality data, including deaths by suicide and external deaths of undetermined intent classified in accordance with the ICD-10. X60-X84 suicide deaths and Y10-Y34 deaths of undetermined intent were identified through death certificates with information on sex, age, ethnic group (information available from 2009), geographic distribution, educational level, month, civil status, and method of suicide. Age was grouped in the following 5-year age brackets: 60–64, 65–69, 70–74, 75–79, and 80 plus. Anonymized and unidentifiable mortality data from INEC are freely available to the public to download on their website.

### Bias

To address the potential source of bias that could derive from underreporting of suicide deaths, we also calculated external deaths of undetermined intent, given that deaths that are not classified as suicides, could be reported in these categories. These were deaths of undetermined intent classified by ICD-10 as Y10-Y34, which include a variety of methods, including poisoning, hanging, firearms use, injury with sharp objects, among others.

### Statistical Analyses

Statistical software package Stata version 15.1 was used to assist in the data analysis. Annual rates per 100,000 of male and female older adults (aged 60 and older) suicide and undetermined deaths were calculated. Rates were calculated for annual age-specific rates for each 5-year age group, based on the WHO standard population and crude rates. To examine the differences in rates, 95% confidence intervals (CIs) were calculated using a Poisson distribution and normal approximation and represented as bars in some figures. Chi-square tests were used to determine the differences between sociodemographic variables by gender.

We used the Joinpoint Regression Program 4.8.0.1, developed by the National Cancer Institute of the United States, to estimate temporal mortality trends (11). The program uses Joinpoint regression to select the simplest model, which identifies significant changes in the trend over the years. The permutation test allows 4,499 permutations to recognize the exact point of a significant change in temporal trends, using the Bonferroni correction for multiple testing with the *P*-value significance set below 0.05 (11). Between each inflection point, the annual percentage changes (APCs) are calculated within the best-fitting model as well as the average annual percentage change (AAPC) for each group.

### Ethical Considerations

This study reports on anonymized and unidentifiable data freely available to the public. Following the Helsinki declaration (12), international and national guidelines of good practice, we used this anonymous database to report on suicide trends ensuring that no harm was caused, or confidentiality breeched.

## Results

Between 1997 and 2019, there were 2,098 suicide deaths in older adults living in Ecuador. Most of the deceased were men 84.0% (*n* = 1,762) and 16.0% (*n* = 336) were women. The majority of the deceased were married or in a relationship (48.6%), and most had reached up to primary level education (46.9%). Ethnicity was recorded from 2009 (*n* = 1,343), and mixed-race mestizos represented the majority of deaths across both men and women (73.1 and 64.2% respectively). Table 1 reports on the sociodemographic characteristics of the deceased reported by sex. Suicide deaths among indigenous people were higher for women (14.4%) when compared to men (4.8%).

**Table 1:** Sociodemographic characteristics and methods of the suicide of people who died by suicide by sex.

**Sociodemographic characteristics**	**Males (*n* = 1,762)**	**Females (*n* = 336)**	**Total (*n* = 2,098)**	**X^**2**^**
Age range				
60–64 65–69 70–74 75–79 80 plus	545 (30.9%) 388 (22.0%) 317 (18.0%) 222 (12.6%) 290 (16.5%)	109 (32.4%) 66 (19.64%) 72 (21.43%) 37 (11.0%) 52 (15.5%)	656 (31.2%) 454 (21.7%) 389 (18.5%) 259 (12.4%) 342 (16.3%)	0.48
Civil status				
Married or in relationship Single Widowed Separated or divorced Missing info	881 (50.0%) 528 (30.0%) 174 (9.9%) 141 (8.0%) 38 (2.1%)	138 (41.1%) 80 (23.8%) 73 (21.7%) 35 (10.4%) 10 (3.0%)	1019 (48.6%) 608 (29.0%) 247 (11.8%) 176 (8.4%) 48 (2.3%)	<0.001
Education				
None Primary level Secondary level Third level or more Missing info	339 (19.2%) 908 (51.5%) 322 (18.3%) 71 (4.0%) 122 (6.9%)	129 (38.4%) 130 (38.7%) 40 (11.9%) 8 (2.4%) 29 (8.6%)	468 (22.3%) 1038 (49.5%) 362 (17.3%) 79 (3.8%) 151 (7.2%)	<0.001
Literacy level				
Can read Cannot read Missing info	1387 (78.7%) 315 (17.9%) 60 (3.4%)	195 (58.0%) 129 (38.4%) 12 (3.6%)	1582 (74.4%) 444 (21.2%) 72 (3.4%)	<0.001
Region				
Sierra/highlands Coast and insular region Amazon	760 (43.1%) 939 (53.3%) 63 (3.6%)	231 (68.8%) 92 (27.4%) 13 (3.9%)	991 (47.2%) 1031 (49.1%) 76 (3.6%)	<0.001
Area				
Urban Rural	1358 (77.0%) 404 (23.0%)	227 (67.6%) 109 (32.4%)	1585 (7.5%) 513 (24.5%)	<0.001
Ethnicity^*^				
Mixed race: mestizo Black White Indigenous Other Missing info	824 (73.1%) 137 (12.1%) 14 (1.2%) 54 (4.8%) 29 (2.6%) 70 (6.2%)	138 (64.2%) 28 (13.0%) 3 (1.4%) 31 (14.4%) 1 (0.5%) 14 (6.5%)	962 (71.6%) 165 (12.3%) 17 (1.3%) 85 (6.3%) 30 (2.2%) 84 (6.3%)	<0.001
Suicide methods				
Intent. drug overdose (X60-X64) Poisoning (X65-X69) Self-cutting (X78) Hanging (X70) Drowning (X71) Firearms (X72-X74) Jumping (X80-X81) Other (X75-X77, X79, X82-X84)	15 (0.9%) 437 (24.8%) 36 (2.0%) 986 (56.0%) 15 (0.9%) 204 (11.6%) 25 (1.4%) 44 (2.5%)	7 (2.1%) 109 (32.4%) 6 (1.8%) 183 (54.5%) 6 (1.8%) 8 (2.4%) 9 (2.7%) 8 (2.4%)	22 (1.1%) 546 (26.0%) 42 (2.0%) 1169 (55.7%) 21 (1.0%) 212 (10.1%) 34 (1.6%) 52 (2.5%)	<0.001

* *Ethnicity only recorded since 2009 (n = 1,343)*.

### Rates by Sex

**Figure 1:**
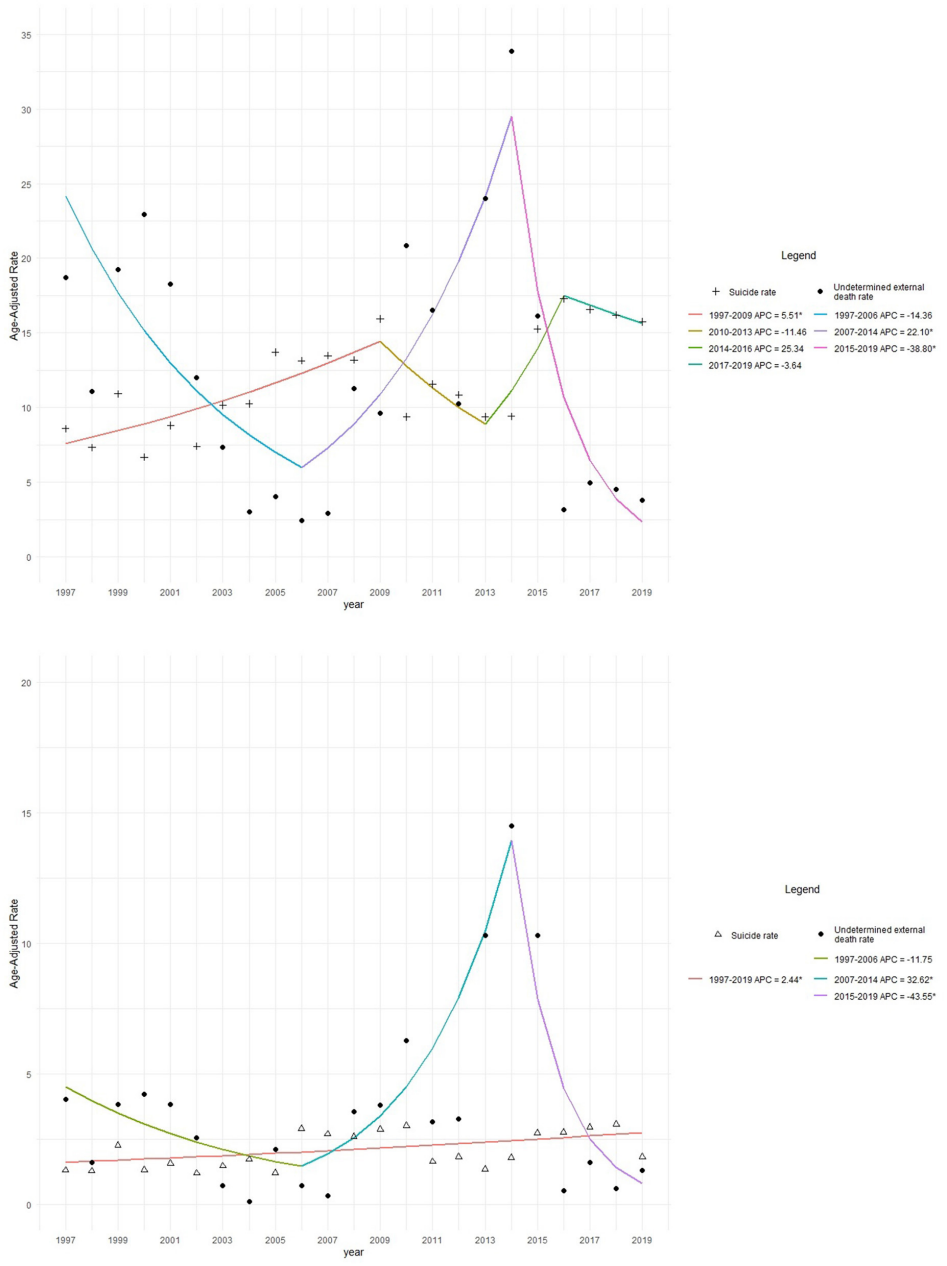
Joinpoint analysis. Age-adjusted suicide and undetermined death (UD) rates in older adults by sex among men and women aged 60 years and older in Ecuador, 1997–2019. **(A)** Age-adjusted suicide and undetermined death (UD) rates in men aged 60 years and older in Ecuador, 1997–2019. **(B)** Age-adjusted suicide and undetermined death (UD) rates in women aged 60 years and older in Ecuador, 1997–2019.

Female suicide rates have remained relatively stable since 1997 in Ecuador, while male rates have fluctuated, with high increases between 2002 and 2009, and 2014 and 2016, and maintained relatively stable within the last 3 years (2017–2019) (as shown in Figure 1). When adding deaths of undetermined intent, increases were noted for both men and women, with sharper increases noted among men (as shown in Figure 1). Undetermined deaths decreased in the early 2000s but increased once again from 2010 onward.

The annual age-adjusted male suicide rate was 29.8 per 100,000, while the female suicide rate was 5.26 per 100,000 during the study period (1997–2019). When adding undetermined deaths, the annual male and female suicide and the undetermined death rate was 60.5 and 14.3 per 100,000, respectively, in 1997–2019.

### Age Differences

Close to a third of participants, 31.2% (*n* = 654) were aged 60–64 years, followed by 21.7% (*n* = 454) aged between 65 and 69 years. In addition, 18.5% (*n* = 389) were 70–74 years, 75–79 years (*n* = 259, 12.4%), and 16.3% (*n* = 342) were 80 years or older. The highest suicide rates were found among the 80 plus age-group, while the lowest suicide rates among the 65–69 age group were in men (refer to Supplementary File 1). Among women, the highest rates were reported in the 70–74 age group, and the lowest rates in the 65–69 age group.

### Rates by Region and Province

**Figure 2:**
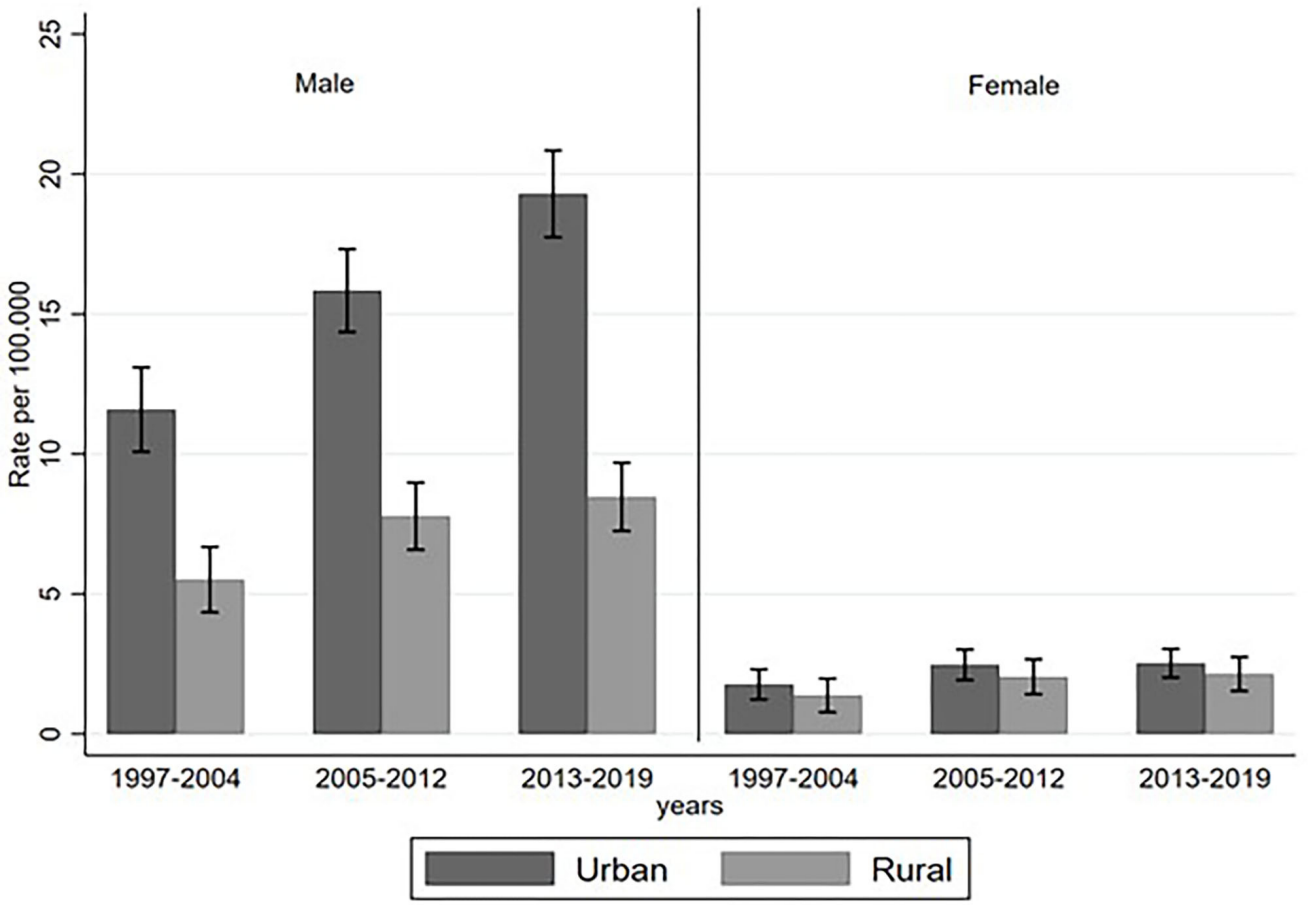
Suicide rates by sex and area type among over 60-year-olds in Ecuador, 1997–2019.

Suicide rates by area (rural and urban) were higher among urban areas in both men and women. Among men, higher suicide rates were observed among those living in urban areas as seen in Figure 2. In terms of provinces, men from Cañar (20.4 per 100,000), Azuay (17.6 per 100,000), and Los Ríos (17.2 per 100,000) had the highest suicide rates during the study period. These provinces are mostly from the Highlands/Sierra (*n* = 2), with Los Ríos representing the Coast. The lowest male suicide rates were reported in the Insular and Coastal regions of Galapagos (0 per 100,000) and Santa Elena (4.3 per 100,000) (refer to Supplementary File 2 for further information). Among women, the highest rates were reported in the Highlands/Sierra: Chimborazo (4.4 per 100,000) and Cotopaxi (4.2 per 100,000) and the lowest rates were consistent with men: Galapagos (0 per 100,000) and Santa Elena (0 per 100,000).

### Differences in Trends

Joinpoint analysis revealed that the AAPC in suicide rates among men was 3.3 (*p* = 0.3) and 2.4% (*p* < 0.05) in women. Among older women, there has been a slight, but constant and significant increase by 2.4% (*p* < 0.05) during 1997–2019. Among older men, there were different periods: between 1997 and 2009, each year suicide rates increased by 5.5% APC (*p* < 0.05), while during 2009–2013, a non-significant decrease of 11.5% APC (*p* = 0.2) was observed. During 2013–2016, another nonsignificant increase was observed (25.3%, *p* = 0.2) and between 2016 and 2019, there was a reduction of 3.6% (*p* = 0.6) (as shown in Figure 1).

In women, while suicide rates seem to be constant, but slowly rising, undetermined deaths between 2006 and 2014 rose by 32.6% yearly (*p* < 0.05). During the same period, in men undetermined deaths increased by 22.2% (*p* < 0.05), while suicides decreased. Undetermined death trends were the same in older women and men: between 1997 and 2006, there was an important, but nonsignificant decrease, while the increase in undetermined deaths during 2006–2015 was statistically significant for both sexes, followed by a sharp and significant decrease during 2014–2019 (as shown in Figure 1).

### Suicide Methods

Across the study period, the most common suicide method was hanging (55.7%) followed by self-poisoning (26.0%) and firearms (10.1%) as reported in Table 1, representing over 90% of suicides by those three methods. Suicide methods remained consistent across males and females, although self-poisoning was more common in a woman (32.4%) = vs. men (24.8%). Self-poisoning included ICD-10 deaths X65-X69, which were chemical substances, as well as alcohol, gases and vapors, and pesticides. While suicide with firearms was more frequent in men 11.6 vs. 2.4%, intentional drug overdose (X 60-X64) was less common (1.1%), but more frequent in women (2.1%) than in men (0.9%).

**Figure 3:**
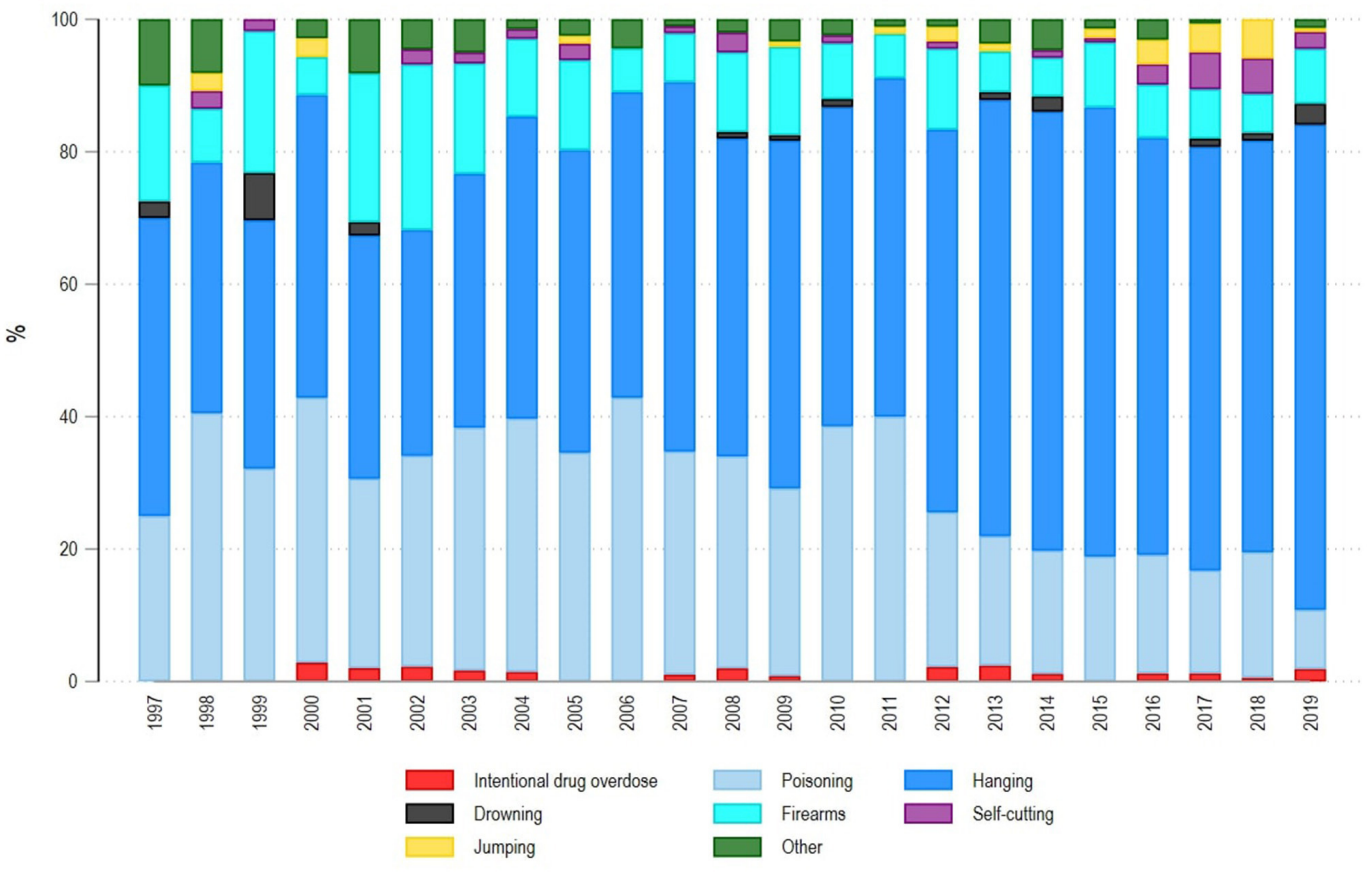
Suicide rates by method among over 60-year-olds in Ecuador, 1997–2019.

Figure 3 shows suicide shifting in suicide methods during 1997–2019. Until 2011, self-poisoning represented 35% of suicides, while between 2012 and 2019, this accounted for only 18%. Suicide in older adults by firearms was more common in 1997–2005 (16%), while since 2006 this accounted for only 8%.

## Discussion

This study is the first to report on suicide and undetermined death rates on adults aged 60 years and older during a 23-year study period: 1997–2019. While research increasingly focuses on suicide among older adults, little is known about the epidemiology and characteristics of suicide rates among older adults in LAMICs, such as Ecuador. During the study period, there were 2,098 suicide deaths in older adults living in Ecuador. Female suicide rates remained relatively stable between 1997 and 2019, while male suicide rates increased between 2002 and 2009, and 2014 and 2016. The annual male suicide and the undetermined death rate was 60.5 per 100,000 in 1997–2019, while the same rate was 14.3 for females. Even though deaths of undetermined intent are difficult to interpret, we might suspect an important number of unregistered deaths of suicide for both sexes between 2006 and 2014, while findings show that since 2014, undetermined deaths have decreased significantly for men and women. This may be an indication of improved quality of the recording of suicides in recent years and improved accuracy of suicide data. The most common suicide method was hanging (55.7%) followed by self-poisoning (26.0%). The highest suicide numbers were reported in urban districts, males, and those with lower education status.

### Comparison With Previous Research

Previous research conducted in Ecuador has shown that suicides among adolescents and young adults (aged 15 to 24) have increased over the past years (2, 3). One previous study reported suicide rates across all age groups in Ecuador from 2001 to 2015 and found that older adults aged 65 and above had a suicide rate of 80 per 100,000, with all other age groups (13- to 20-year old's, 21- to 39-year old's, and 40- to 64-year old's) having higher rates (2). Our research indicates that suicide rates among older men have increased over the past decade, while suicide rates among older women have remained relatively stable over the years.

International research indicates that suicide rates in older adults vary. In South Korea, suicide rates among older adults aged 65 and older have been increasing from 2000 to 2011 with male suicide rates increasing from 55.6 to 128.6 per 100,000 and, 23.6 to 46.1 per 100,00 among women (13). Similarly, in Brazil, suicide rates have been increasing from 1997 to 2015 among men and women aged 60 years and older with a mean suicide rate of 13.8 per 100,000 among men and 2.6 per 100,000 among women (14). However, in Japan, rates of suicide among older adults aged 65 and older have decreased between 2000 and 2011, with male suicide rates decreasing from 46.8 to 38.5 per 100,000 and 23.3 to 18.6 per 100,000 among women (13). In Ireland, previous research suggests suicide rates to be 22.1 per 100,000 among men and 7.6 per 100,000 among women, which indicates an overall increase over the past few years (15).

Previous research conducted on suicide rates of older adults for each country listed in the United Nations Statistics Division showed that suicide rates increase with age from 60–64 to 65–69 and further 5-age bands until 95–99 years among both men and women (16).

Suicide among older men in Ecuador increased between 1997 and 2006. Possible reasons for this increase could be related to the financial crisis Ecuador went through during those years which started in 1999 and forced over 1 million Ecuadorians to migrate and leave their children with their grandparents as caregivers (17). Previous research has shown that financial crises affect mostly suicide among men when compared to suicide among women, which is also consistent with our findings (18).

Our research found that suicide rates among urban older men increased. Between 1997 and 2004, male suicide rates in urban areas were two times as high compared to rural areas, while between 2013 and 2019, urban suicide rates were nearly three times higher when compared to rural suicide rates among men. In rural areas, further social support and structure may exist when compared to urban areas, which contribute to a sense of belongingness, a protective factor for suicide (19). When examining suicide rates by provinces, findings from our study are consistent with previous research examining suicide rates across individuals of all ages in Ecuador: highest suicide rates reported in provinces from the Sierra/Highlands (Cañar, Azuay, Cotopaxi, and Chimborazo) (2). In the Sierra/Highlands provinces reported, the largest percentage of the indigenous population reside; therefore, this could be an indication of increased risk of suicide and indigenous status. Furthermore, suicide deaths among indigenous people were higher among females (14.4%) when compared to males (4.8%). Previous research conducted in Australia and New Zealand examining suicide among indigenous populations showed that most indigenous female suicides occur among younger women (20, 21). To the best of our knowledge, no previous research has examined suicide rates among indigenous older adults in Ecuador. However, research from Brazil indicates that there is an increased risk of suicide among indigenous older adults with no income or paid activity and females (22).

In relation to suicide methods, our findings show an increase among hanging, while suicide due to self-poisoning and firearms has decreased over the last decade. This could be due to an increasing governmental and societal awareness and public health measures relating to suicide, specifically pesticide control among farmers (23). Another possible explanation is the improvement in care to people presenting with intentional drug overdose or self-poisoning as the Centre for Information and Advice in Toxicology (CIAT) was established in 2008. CIAT provides 24/7 telephone service to healthcare professionals and citizens calling 911 (emergency number) following an overdose or self-poisoning in order to receive support by emergency services if needed. Previous research shows that public and community-wide regulations which prioritize limited access to pesticides are effective interventions to treat suicides due to self-poisoning (24). Furthermore, the establishment of toxicology centers is a commitment of all the states belonging to the WHO, which recognizes the impact on the management of poisonings, whether intentional or accidental. Evidence also shows that restrictive gun laws contribute to reducing suicide in older adults (25).

### Strengths and Limitations

This is the first national study that reports on full coverage of suicide rates and deaths of undetermined intent among older adults aged 60 and older living in Ecuador, examining these rates further among 5-year age groups (e.g., 60–64, 65–69, 70–74, 75–79, and 80 plus). This study reports on suicide deaths of older adults living in Ecuador during a 23-year study period: 1997–2019 based on information from the official national mortality database: The National Institute of Census and Statistics (8). In light of international recognized priorities, including the WHO (Global Mental Health Action Plan) and United Nations (Sustainable Development Goals, Goal 3), this research provides further evidence and insight of reducing rates of suicides.

Among the study limitations are issues relating to late registration of suicide and undetermined deaths. Late registered deaths may exist or lower levels of registers due to problems with official registries. While including 23 years of data is considered a strength of our study, findings must be interpreted with caution due to the varying reporting of suicide and undetermined deaths, as well as the quality of this reporting. Furthermore, stigma is a contributing factor to underreporting of suicide deaths worldwide, but also in Ecuador. Suicide-related stigma could have caused underreporting of suicide due to family members requesting death certificates to be changed to death of unknown intent. Therefore, the data reported in this study could represent an underreporting of suicide deaths among older adults in Ecuador.

### Conclusions and Implications

The findings from this study highlight priority groups among older adults living in Ecuador at risk of suicide. The study contributes to building the baseline for further studies on suicide rates of older adults living in Ecuador and the context of the UN SDGs. Older males, older adults living in urban districts, and those with lower education status had the highest suicide rates. Hanging continues to be the most common method for suicide. Results from this study highlight priority areas for suicide prevention in older adults such as limited access to means, responsible media reporting of suicide methods, and mental health promotion activities. By examining suicide trends over 23 years, our findings can help inform policy and future interventions targeting suicide prevention among at-risk groups, including older adults.

## Data Availability Statement

Publicly available datasets were analyzed in this study. This data can be found here: https://aplicaciones3.ecuadorencifras.gob.ec/BIINEC-war/index.xhtml.

## Author Contributions

MIT, RG, and EA conceptualized the study with input from the co-authors. FN conducted the statistical analysis with support of RG. MIT and RG wrote the first draft with input received from all co-authors. All the authors are guarantors for the study. All authors revised the manuscript, provided critical scholarly feedback, and approved the final version of the manuscript. The corresponding author attests that all listed authors meet authorship criteria and that no others meeting the criteria have been omitted.

## Funding

EA and MIT are supported by the Irish Health Research Board (Grant Number: IRRL-2015-1586). The funding source had no involvement in study design, collection, analysis, and interpretation of data, writing of the report, or in the decision to submit the article for publication.

## Conflict of Interest

The authors declare that the research was conducted in the absence of any commercial or financial relationships that could be construed as a potential conflict of interest.

## Publisher's Note

All claims expressed in this article are solely those of the authors and do not necessarily represent those of their affiliated organizations, or those of the publisher, the editors and the reviewers. Any product that may be evaluated in this article, or claim that may be made by its manufacturer, is not guaranteed or endorsed by the publisher.
