# Supplementary material for: Sociodemographic Analysis of Suicide Rates Among Older Adults Living in Ecuador: 1997–2019
Source: Front Public Health. 2021 Oct 8;9:726424. doi: 10.3389/fpubh.2021.726424 (PMC8531474; doi:10.3389/fpubh.2021.726424)
Supplement: Supplementary file 1 [file Data_Sheet_1.docx]

# Supplementary Materials

Supplementary File 1. Suicide rates by sex and age among over 60-year-olds in Ecuador, 1997-2019


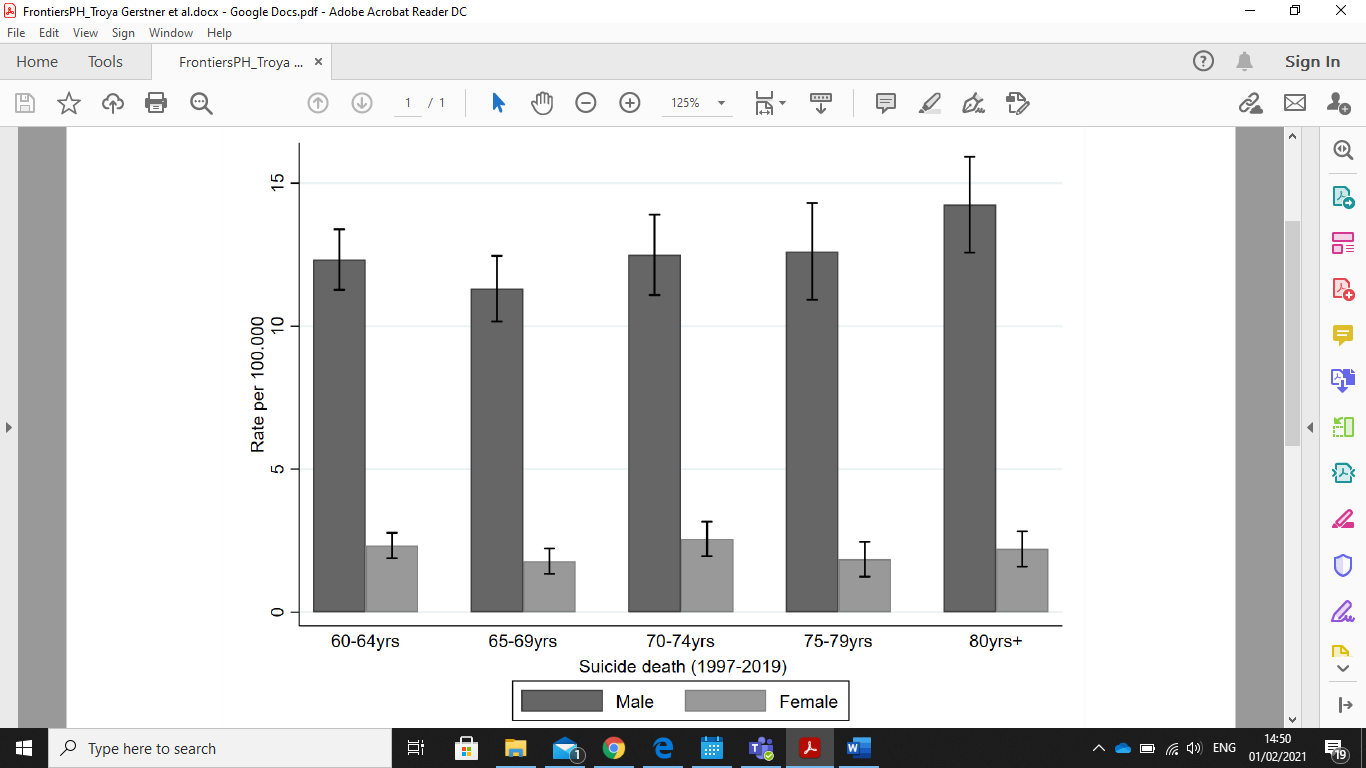


Supplementary File 2. Suicide rates by province in over 60-year-olds in Ecuador, 1997-2019

|  | Male | | Female | |
| --- | --- | --- | --- | --- |
| Province | No. of suicides | Rate | No. of suicides | Rate |
| Azuay | 125 | 17.57 | 37 | 3.86 |
| Bolivar | 36 | 14.94 | 9 | 3.51 |
| Cañar | 51 | 20.37 | 11 | 3.41 |
| Carchi | 32 | 15.80 | 6 | 2.70 |
| Cotopaxi | 55 | 12.81 | 21 | 4.24 |
| Chimborazo | 83 | 15.73 | 29 | 4.39 |
| El Oro | 99 | 14.53 | 9 | 1.53 |
| Esmeraldas | 51 | 10.99 | 5 | 1.15 |
| Guayas | 431 | 12.60 | 38 | 1.01 |
| Imbabura | 47 | 10.80 | 18 | 3.51 |
| Loja | 53 | 9.13 | 11 | 1.77 |
| Los Ríos | 131 | 17.22 | 9 | 1.31 |
| Manabí | 173 | 12.53 | 24 | 1.67 |
| Morona | 12 | 11.51 | 4 | 3.90 |
| Napo | 11 | 16.03 | 1 | 1.50 |
| Pastaza | 10 | 15.91 | 2 | 3.44 |
| Pichincha | 197 | 8.33 | 65 | 2.26 |
| Tungurahua | 81 | 13.95 | 24 | 3.52 |
| Zamora | 6 | 7.63 | 0 | 0.00 |
| Galápagos | 0 | 0.00 | 0 | 0.00 |
| Sucumbíos | 13 | 10.31 | 4 | 4.13 |
| Orellana | 11 | 15.25 | 2 | 3.38 |
| Santo Domingo | 42 | 13.41 | 7 | 2.41 |
| Santa Elena | 12 | 4.34 | 0 | 0.00 |
